# IL-17-differentiated macrophages secrete pro-inflammatory cytokines in response to oxidized low-density lipoprotein

**DOI:** 10.1186/s12944-017-0588-1

**Published:** 2017-10-10

**Authors:** María de la Paz Sánchez-Martínez, Francisco Blanco-Favela, Mónica Daniela Mora-Ruiz, Adriana Karina Chávez-Rueda, Mariela Bernabe-García, Luis Chávez-Sánchez

**Affiliations:** 1 Unidad de Investigación Médica en Inmunología, Hospital de Pediatría, Centro Médico Nacional Siglo XXI, Instituto Mexicano del Seguro Social, Av. Cuauhtémoc 330 Col. Doctores, 06720 Ciudad de México, Mexico; 2 Unidad de Investigación Médica en Nutrición, Hospital de Pediatría, Centro Médico Nacional Siglo XXI, Instituto Mexicano del Seguro Social, Av. Cuauhtémoc 330 Col. Doctores, 06720 Ciudad de México, Mexico

**Keywords:** Interleukin-17, Macrophages, Oxidized-low density lipoprotein

## Abstract

**Background:**

Cytokines and macrophages play a central role in the development of atherosclerosis. Interleukin (IL)-17 is a pro-inflammatory cytokine with differential effects on innate immune cells. We investigated the effects of IL-17 on macrophage differentiation and foam cell formation and activation in response to oxidized low-density lipoprotein (oxLDL).

**Methods:**

Human monocytes were treated with IL-17 to induce macrophage differentiation. As controls, human monocytes were differentiated into M1 macrophages (M1) or M2 macrophages (M2). Subsequently, we analyzed the expression levels of markers such as CD80, CD36 and Toll-like receptors (TLRs) as well as foam cell formation and cytokines in M1, M2 and macrophages differentiated with IL-17 with or without oxLDL.

**Results:**

The expression of M1 or M2 markers or cytokines was not induced in macrophages differentiated with IL-17. Macrophages differentiated with IL-17 formed few foam cells, with an average proportion of 20%, and expressed 3 times as much TLR2 and 3.8 times as much TLR4 as M0 macrophages. Additionally, macrophages differentiated with IL-17 acquired inflammatory capacity in response to oxLDL through the expression of specific markers, such as CD80, which increased 18-times compared with macrophages differentiated with IL-17 alone, and secreted 1.3 times less tumor necrosis factor (TNF)-α than M1. Additionally, oxLDL increased the levels of CD80, CD86 and IL-6 by 5.7, 2.8 and 1.4 times in M1 compared with M1 in the absence of oxLDL. In M2, oxLDL induced increases in the secretion of IL-6 and TNF-α that were 1.9 times and 1.2 times smaller, respectively, than those observed in M1.

**Conclusion:**

Our study demonstrates that differentiation of macrophages with IL-17 does not induce the expression of markers or cytokines characteristic of M1 or M2 and these macrophages form few foam cells; however, the expression of TLR is increased. Moreover, these macrophages acquire the inflammatory capacity as evidenced by the expression of costimulatory molecules and secretion of pro-inflammatory cytokines in response to oxLDL. These findings suggest that the activation of macrophages differentiated with IL-17 by oxLDL contributes to the inflammatory process of atherosclerosis.

## Background

The interleukin (IL)-17 family has six members designated A-F; IL-17A (subsequently referred to as IL-17) is the most studied isoform [1]. Several types of cells secrete IL-17, such as CD8^+^ T cells, natural killer cells, and T-helper 17 (Th17) [1–3]. The role of IL-17 has been studied in chronic inflammatory diseases, such as autoimmune disease and atherosclerosis [1, 4, 5].

The development and progression of atherosclerotic lesions are characterized by an inflammatory response and accumulation of oxidized low-density lipoprotein (oxLDL) [6, 7]. Macrophages are recognized as a major player in atherosclerosis through the induction of inflammation and foam cell formation in response to oxLDL [6–8]. In atherosclerotic lesions, macrophages respond to various environmental stimuli, such as cytokines, which can modify their phenotypes, such as M1 and M2 [9, 10]. Thus, interferon (IFN)-γ, in combination with lipopolysaccharide (LPS) and granulocyte macrophage-colony stimulating factor (GM-CSF), induces M1 macrophages (M1), which are characterized by the secretion of high levels of tumor necrosis factor (TNF)-α and IL-6 and low levels of IL-10. IL-4 induces the development of M2 macrophages (M2), which secrete high levels of IL-10 and low levels of pro-inflammatory cytokines, such as TNF-α [8, 11]. Both types of macrophages are present in both human and mouse atherosclerotic lesions [9, 12, 13]. Functionally, M1 and M2 have been suggested to promote and resolve plaque inflammation, respectively [8, 10].

The microenvironment of atherosclerotic plaques is complex and may be influenced by several types of cells via the secretion of cytokines, such as IL-17. Atherogenic LDb or Apo E^−/−^ mice have increased numbers of IL-17- and Th17-positive cells in atherosclerotic lesions, which were associated with an increased size of atherosclerotic plaques in mice [14, 15]. Treatment of ApoE^−/−^ mice with neutralizing anti–IL-17 antibody inhibited the development of atherosclerotic plaques, which was accompanied by a decreased number of macrophages in the lesion [15]. Similarly, IL-17ra^−/−^ mice have decreased monocytes, macrophages, and inflammatory responses [16]. These results suggest that IL-17 contributes to atherosclerosis, as well to the development of monocytes/macrophages. Additionally, previous in vitro studies have demonstrated that IL-17A polarizes macrophages toward a pro-inflammatory transcriptome and upregulates cytokines, such as IL-6 and CCL2 [17], as well as IL-1β and TNF-α [18].

These findings suggest that IL-17 participates in the differentiation and activation of macrophages. Despite these findings, the mechanisms involved in foam cell differentiation and the production of molecules involved in the inflammatory response by IL-17 in macrophages are complex and have not been fully characterized. Accordingly, we analyzed the involvement of IL-17 in human macrophages by assessing expression of markers associated with M1 or M2 phenotypes, foam cell differentiation and pro-inflammatory cytokine secretion in response to oxLDL.

## Methods

### Experimental protocol

Informed consent was obtained from ten healthy male and female normolipidemic volunteers who ranged from 20 to 30 years of age. The volunteers had no cardiovascular risk factors or clinically apparent atherosclerotic disease. The study was approved by the Human Ethics Committee and Medical Research of the Mexican Social Security Institute and was conducted according to the guidelines of the Declaration of Helsinki.

### LDL isolation and modification

Human LDL was isolated from normolipidemic plasma by density ultracentrifugation and dialyzed against phosphate-buffered saline (PBS)/0.5 mM EDTA (Sigma-Aldrich, St. Louis, MO, USA). EDTA was removed prior to oxidation by extensive dialysis, and oxLDL was prepared by incubation of 300 μg/ml of low-density lipoprotein (LDL) with 10 mM CuSO_4_ for 18 h at 37 °C. The degree of oxLDL oxidation was determined using thiobarbituric acid-reactive substances [19]. All LDL preparations used in these experiments were tested for bacterial LPS contamination using a Limulus Amoebocyte Lysate kit (BioWhittaker, Walkersville, MD, USA) according to the manufacturer’s instructions.

### Monocyte isolation

Peripheral blood mononuclear cells (PBMCs) were obtained from healthy volunteers by density centrifugation using Lymphoprep (Axis-Shield, Oslo, Norway). The blood samples were mixed with an equal volume of PBS, pH 7.4, layered over 3 ml of Lymphoprep, and centrifuged at 700 x g for 30 min. The recovered PBMCs were washed three times with PBS at pH 7.4. The monocytes were then isolated from PBMCs by negative selection (Pan Monocyte Isolation Kit, Miltenyi Biotec, Bergisch Gladbach, Germany). PBMCs were incubated with FcR Blocking Reagent and a cocktail of biotin-conjugated antibodies against antigens that are not expressed on human monocytes. Magnetic microbeads were coupled to an anti-hapten monoclonal antibody and depleted using a magnetic column. The entire effluent was collected and identified as the monocyte-enriched fraction. Purified cells were stained for CD14, and the purity of monocytes was >90% as determined by flow cytometry.

### Differentiation of monocytes into macrophages

The monocytes (4 × 10^5^/ml) were cultured in RPMI 1640 medium supplemented with 2 mM glutamine, antibiotics and 10% fetal calf serum in the presence of 40 ng/ml recombinant human (rh) GM-CSF (rhGM-CSF was donated by Probiomed, México City, México) for M1 or 50 ng/ml of rh macrophage colony-stimulating factor (M-CSF) (*R&D* Systems, Minneapolis, MN, USA) for M2 for 5 days (the culture medium was replaced every 2 days with fresh culture medium containing rhGM-CSF or rhM-CSF). For complete polarization, M1 were stimulated with 100 ng/ml LPS (Sigma-Aldrich, St. Louis, MO, USA) and 25 ng/ml IFN-γ (*R&D* Systems, Minneapolis, MN, USA), and M2 were cultured with 10 ng/ml IL-4 and 40 ng/ml IL-13 (*R&D* Systems, Minneapolis, MN, USA) for 24 h. In contrast, monocytes were cultured with 60 ng/ml rhIL-17 (*R&D* Systems, Minneapolis, MN, USA) for 6 days (the culture medium was replaced with fresh culture medium containing rhIL-17 every 2 days) to obtain IL-17 differentiated macrophages. For M0 macrophages (M0), monocytes were cultured only in culture medium.

### Flow cytometry

M1 were stained with anti-CD80, anti-CD86, anti-TLR2 and anti-TLR4 antibodies (eBioscience, San Diego, CA, USA), and M2 were stained with anti-CD36, anti-CD206, anti-TLR2 and anti-TLR4 antibodies. Macrophages polarized with IL-17 were stained with anti-CD80, anti-CD86, anti-CD36, anti-CD206, anti-TLR2 and anti-TLR4 antibodies. M0 were stained with the same antibodies used for M1, M2 and TLR. All antibody staining was performed for 20 min in the dark at 4 °C; the cells were then washed twice with PBS containing 1% bovine serum albumin and 1% sodium azide (Sigma-Aldrich, St. Louis, MO, USA). In all assays, dead cells were identified using Zombie (Biolegend, San Diego, CA, USA). Expression levels were measured using a MACSQuant flow cytometer (Miltenyi Biotec, Bergisch Gladbach, Germany) and quantified based on the mean fluorescence intensity (MFI) of each sample using FlowJo V10 software (Tree Star, San Carlos, CA, USA).

### Assessment of foam cell formation

M1, M2, M0 and macrophages polarized with IL-17 were stimulated with 30 μg/ml oxLDL for 24 h at 37 °C in Lab Tek II chamber slides (Nalge Nunc). Next, the medium was aspirated, and the cells were washed twice with PBS, fixed for 10 min in 2% paraformaldehyde (in PBS), rinsed in 60% isopropanol for 15 s, and stained for 10 min in 3% Oil Red O (in 60% isopropanol) in the dark. The cells were then washed with 60% isopropanol and counterstained with hematoxylin for 2 min, followed by washing three times with PBS. Solutions were freshly prepared and used within 1 h of preparation. The designation of a macrophage as a foam cell required positive Oil Red O staining. The foam cells were examined by light microscopy (magnification, ×40; Axiolab HBO microscope; Nikon, Garden City, NY, USA) [20].

### Cytokine analysis

Cytokines were measured in culture supernatants of M0, M1, M2 and macrophages differentiated with IL-17 treated with or without 30 μg/ml of oxLDL by ELISA (eBioscience, San Diego, CA, USA) according to the manufacturer’s instructions.

### Statistical analysis

All experiments were carried out in at least in duplicate. Each group consisted of ten human subjects. Data are presented as the mean ± SEM. Statistical assessment was performed with one-way analysis of variance followed by Kruskal Wallis test with the GraphPad Prism 5 software package (GraphPad, La Jolla, CA, USA). Differences were considered statistically significant at *P* < 0.05.

## Results

### Expression of M1 and M2 marker-related phenotypes on macrophages differentiated with IL-17

**Fig. 1:**
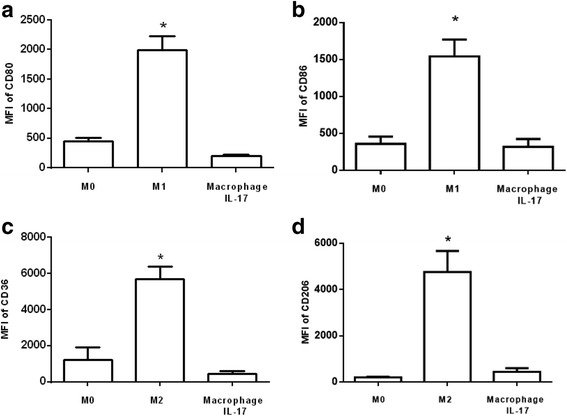
Phenotypes of M1, M2 and macrophages polarized with IL-17. The expression levels of **a** CD80 and **b** CD86 were determined in M1 and M17, and the expression levels of **c** CD36 and **d** CD206 were assessed in M2 and M17. The expression levels are expressed as the mean fluorescence intensity (MFI). The results represent ten independent experiments performed in duplicate. *p < 0.05

We evaluated the expression levels of M1 and M2 markers on macrophages differentiated with IL-17. M1 expressed high levels of CD80 and CD86 (9.1 and 4.1 times higher, respectively, than that expressed by macrophages differentiated with IL-17 and by M0 (Fig. 1a and b). By contrast, M2 expressed higher levels of CD36 (15 times higher) and CD206 (9.8 times higher) than macrophages differentiated with IL-17 and M0 (Fig. 1c and d). Human monocytes differentiated into macrophages in the presence of IL-17 (60 ng/ml) for 6 days expressed similar levels of CD80, CD86, CD36 and CD206 as M0 (Fig. 1a-d). These results suggest that IL-17 does not affect surface molecules related to M1 or M2 phenotypes, which is consistent with previous results [17].

### Role of M1, M2 and macrophages differentiated with IL-17 in the formation of foam cells

**Fig. 2:**
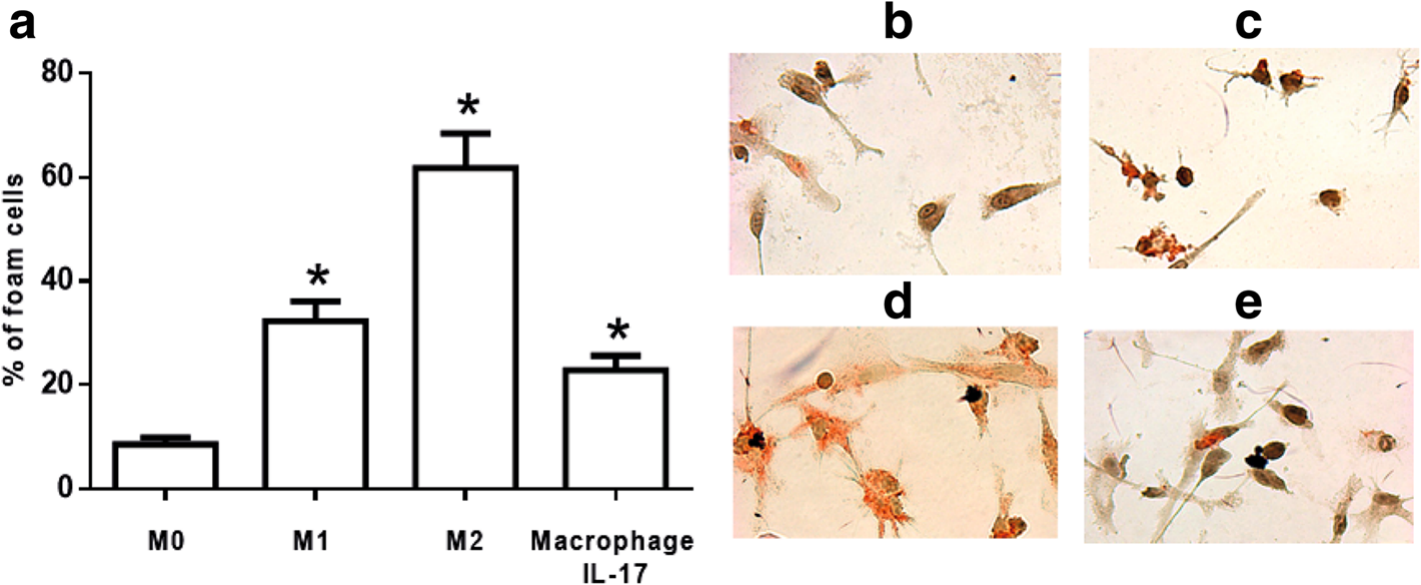
Foam cell formation in M1, M2 and macrophages polarized with IL-17. **a** M0, M1, M2 and macrophages polarized with IL-17 were cultured with oxLDL (30 μg/ml) for 24 h, and the foam cells were quantified. Representative images of foam cells stained with Oil Red O. **b** M0, **c** M1, **d** M2 and **e** macrophages polarized with IL-17. The results represent five independent experiments performed in duplicate. *p < 0.05

Although IL-17 did not induce an increase in the expression of surface molecules related to M1 and M2 in macrophages differentiated with IL-17, we decided to evaluate the formation of foam cells in different types of macrophages. M1 and M2 were cultured with oxLDL (30 μg/ml) for 24 h and then stained with Oil Red O, which revealed that M1 exhibited an average increase in the number of foam cells of 25% (Fig. 2a and c). By contrast, M2 displayed an increase in the formation of foam cells of 70% (Fig. 2a and d). Macrophages differentiated with IL-17 formed few foam cells (20% foam cells), as shown in Fig. 2a and e. These results suggest that compared with M1 and M2, macrophages differentiated with IL-17 participate in the formation of foam cells to a lesser extent.

### Expression levels of TLR2 and TLR4 in M1, M2 and macrophages differentiated with IL-17

**Fig. 3:**
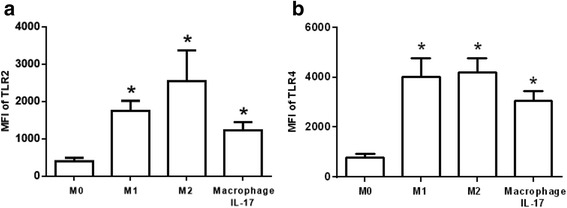
Expression of TLR on M1, M2 and macrophages polarized with IL-17. The expression levels of **a** TLR2 and **b** TLR4 were determined in M0, M1, M2 and macrophages polarized with IL-17. The expression levels are expressed as the mean fluorescence intensity (MFI). The results represent ten independent experiments performed in duplicate. *p < 0.05

IL-17 induces TLR2 and TLR4 expression on fibroblast-like synoviocytes [21]. Therefore, we analyzed the expression levels of TLR2 and TLR4 on M1, M2 and macrophages differentiated with IL-17. TLR2 and TLR4 levels were increased 4.3- and 5-times on M1 compared with M0 (Fig. 3a and b) and 6.3-times and 5.3-times on M2 compared with M0 (Fig. 3a and b). Moreover, macrophages differentiated with IL-17 exhibited increases of TLR2 and TLR4 of 3- and 3.8-times, respectively, compared to M0 (Fig. 3a and b). These results suggest that macrophages differentiated with IL-17 express TLR2 and TLR4, which may contribute to the activation of macrophages.

### Effect of oxLDL on the phenotypes of M1, M2 and macrophages differentiated with IL-17

**Fig. 4:**
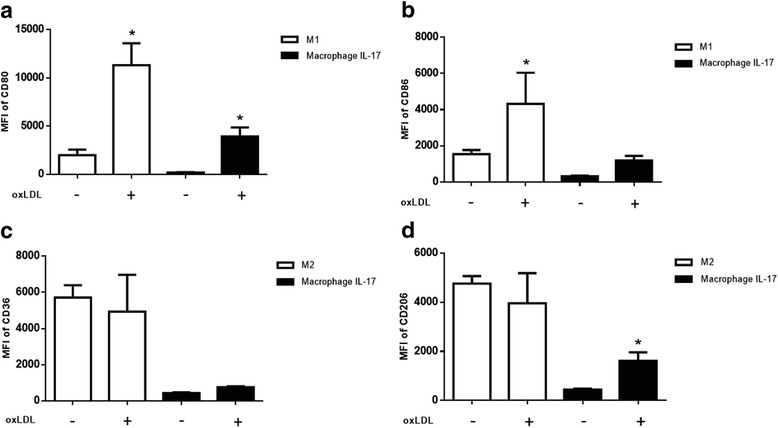
Effect of oxLDL on the phenotypes of M1, M2, and macrophages polarized with IL-17. M1, M2 and macrophages polarized with IL-17 were incubated with 30 μg/ml oxLDL for 24 h at 37 °C. The expression levels of **a** CD80, **b** CD86, **c** CD36 and **d** CD206 were determined by flow cytometry. The results represent ten independent experiments performed in duplicate. *p < 0.05

In atherosclerosis, oxLDL is an atherogenic molecule that induces macrophage activation, which contributes to the development of lesions [6, 7]. We evaluated the expression of markers on M1, M2 and macrophages differentiated with IL-17 in response to oxLDL. Compared with M1 in the absence of oxLDL, M1 stimulated with oxLDL displayed a 5.7-times increase in CD80 levels (Fig. 4a) and a 2.8-times increase in CD86 levels (Fig. 4b). However, no changes in CD36 or CD206 were detected in M2 stimulated with oxLDL compared with M2 in the absence of oxLDL (Fig. 4c and d). These results suggest that oxLDL increases the levels of markers associated with the M1 phenotype and does not induce changes in the M2 phenotype. Meanwhile, compared with macrophages differentiated with IL-17 without oxLDL, macrophages differentiated with IL-17 and treated with oxLDL showed an 18-times increase in CD80 levels and a 3.7-times increase in CD86 levels (Fig. 4a and b). Moreover, a 1.7-times increase in CD36 (Fig. 4c) and a 3.6-times increase in CD206 (Fig. 4d) were detected in macrophages differentiated with IL-17 and treated with oxLDL compared with macrophages cultured only with IL-17. These results suggested that oxLDL increased the levels of CD80 and CD86 in M1 and did not affect markers associated with M2. In addition, oxLDL preferentially induced the expression levels of inflammatory macrophage markers, as well as M2 markers, on macrophages differentiated with IL-17.

### The roles of IL-17 and oxLDL in cytokine production by macrophages

**Fig. 5:**
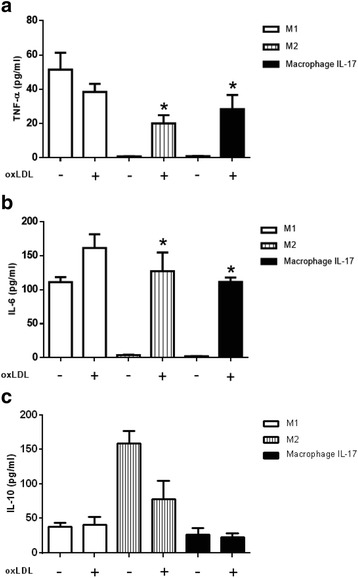
Production of cytokines by M1, M2 and M17 in response to oxLDL. M1, M2 and macrophages polarized with IL-17 were incubated with 30 μg/ml oxLDL for 24 h at 37 °C. The concentrations of **a** TNF-α, **b** IL-16 and **c** IL-10 in culture supernatants were determined by ELISA. The results represent ten independent experiments performed in duplicate. *p < 0.05

Macrophages secrete pro-inflammatory cytokines in response to oxLDL [6, 7]. Thus, we evaluated the effect of oxLDL on the secretion of cytokines in the macrophages. M1 stimulated with oxLDL produced 0.74 times less than the levels of TNF-α that were produced by M1 in the absence of oxLDL (Fig. 5a). Moreover, in the presence of oxLDL, these macrophages produced increased levels of IL-6 (1.4 times higher than that in the absence of this lipoprotein; Fig. 5b). Additionally, the oxLDL stimulus did not increase the secretion of IL-10 in M1 compared with that in M1 without oxLDL (Fig. 5c). On the other hand, oxLDL induced on average the secretion of 20 pg/ml TNF-α by M2 (on average, 1.9 times less than that secreted by M1 with oxLDL), as well as 132.2 pg/ml IL-6 on average (1.2 times less than that secreted by M1 with oxLDL), as shown in Figs. 5a and b, respectively. In the presence of oxLDL, M2 secreted lower levels of IL-10 than in the absence of oxLDL. Moreover, macrophages differentiated with IL-17 were able to secrete on average 30.1 pg/ml TNF-α (1.3 times less than that secreted by M1 with oxLDL), as shown in Fig. 5a. Additionally, macrophages differentiated with IL-17 did not secrete TNF-α or IL-6 (Fig. 5a and b) but secreted low levels of IL-10 similar to that of M1. However, macrophages differentiated with IL-17 secreted on average 30.1 pg/ml TNF-α and 120.3 pg/ml IL-6 (1.3 times and 1.4 times less, respectively, than that secreted by M1 with oxLDL), as shown in Fig. 5b. Additionally, oxLDL did not affect the secretion of IL-10 by macrophages differentiated with IL-17 (Fig. 5c). These results suggest that M1 increase their ability to secrete IL-6 in response to oxLDL. In addition, M2 secrete pro-inflammatory cytokines in the presence of oxLDL, suggesting a conversion from M2 to M1. Macrophages differentiated with IL-17 secreted TNF-α and IL-6 in response to oxLDL but secreted similar levels of IL-10 in the absence or presence of oxLDL.

## Discussion

Atherosclerosis is a chronic inflammatory disease that is characterized by complex interactions among diverse cell types and cytokines. Macrophages are essential for the development of atherosclerosis through the induction of the inflammatory response and the formation of foam cells [6–8]. These functions may be influenced by cytokines such as IL-17, which contributes to the secretion of pro-inflammatory cytokines in macrophages. However, the reported evidence for IL-17 in these cells remains controversial and has not been fully characterized. Here, we demonstrate that IL-17 does not contribute to the differentiation of macrophages. However, in the presence of oxLDL, this cytokine contributes little to the formation of foam cells and plays a relevant role in the secretion of pro-inflammatory cytokines by macrophages differentiated with IL-17.

Macrophages are key in atherosclerosis [6–9], and functional differentiation and polarization are hallmarks of macrophages that result in the phenotypic diversity of the macrophage subsets [22]. M1 typically express increased levels CD80 and CD86, while M2 express increased levels of CD36 and CD206 [9, 10, 22]. Treatment with IL-17 did not affect the expression levels of markers characteristic of M1, such as CD80 and CD86, or M2, such as CD36 or CD206, which suggests that IL-17 did not affect the expression levels of markers related to M1 or M2 phenotypes; previous studies have reported similar results [17].

oxLDL and macrophages play a central role in the formation of foam cells that contributes to the development of atherosclerosis [6, 7]. We found that compared with M1, M2 formed a considerable number of foam cells, which suggests that M2, and to a lesser extent, M1 can contribute to the development of atherosclerosis through the formation of foam cells [17, 23]. In addition, macrophages differentiated with IL-17 contributed to the formation of foam cells to the same extent as M1. IL-17 favors the capture of modified LDL [24] because it increases the expression of scavenger receptors such as macrophage scavenger receptor 1 [25], which can internalize oxLDL [26]. In addition, IL-17 enhances the expression of distinctive markers of foam cells such as the nuclear receptor liver X receptor-α, as well as its target genes, such as ATP-binding cassette transporters A1, apolipoprotein C1 and apolipoprotein E in antigen-presenting cells [25]. This evidence and our results suggest that macrophages differentiated with IL-17 can contribute to the formation of foam cells, albeit to a small degree.

Macrophages are cells of the innate immune system that are activated through TLRs [27]. IL-17 increases the expression levels of TLR2 and TLR4 on fibroblast-like synoviocytes [21]. Here, we showed that macrophages differentiated with IL-17 expressed TLR2 and TLR4, similar to M1 and M2. Our results suggest that macrophages differentiated with IL-17 can be activated by damage-associated molecular patterns such as oxLDL. Consistent with this finding, previous studies have demonstrated that TLR2 and TLR4 contribute to the activation of macrophages to respond to oxLDL [28, 29].

oxLDL is an atherogenic molecule that increases the expression levels of co-stimulatory molecules, such as CD86, in macrophages [29]. We found that M1 expressed higher levels of CD80 and CD86 in response to the oxLDL stimulus than M1 that were not exposed to oxLDL. These results suggest that oxLDL increases the inflammatory capacity of M1, which could contribute to the development of atherosclerotic plaques [30]. Additionally, oxLDL did not modify the expression levels of CD36 and CD206 on M2 compared with those of M2 without oxLDL. In the presence of oxLDL, compared with macrophages polarized with IL-17 that were not stimulated with oxLDL, macrophages differentiated with IL-17 exhibited increased levels of CD80 and CD206 and to a lesser extent, CD86, suggesting that IL-17 favors the expression of M1 and M2 markers on macrophages differentiated with IL-17. In this regard, IL-17 induces the secretion of IL-1β and CD163, which are markers of M1 and M2, respectively [17, 18].

During the pathogenesis of atherosclerosis, macrophages respond to several proatherogenic proteins, such as cytokines and oxLDL [6–8]. In this context, we observed increased secretion of IL-6 by M1 stimulated with oxLDL compared with that by M1 without oxLDL, suggesting that oxLDL increases the inflammatory activity of M1 [30]. M2 secreted TNF-α, IL-6, and IL-10 in response to oxLDL, suggesting that oxLDL induces a conversion from M2 to M1; in this regard, oxLDL has been shown to induce the secretion of IL-8 by M2 [30]. Previous studies have demonstrated the role of pro-inflammatory cytokines in the development of atherosclerotic lesions [6] and suggested that cytokines such as IFN-γ can contribute to the differentiation and activation of macrophages [7, 22, 31]. However, here we found that macrophages cultured only with IL-17 for 6 days secreted low levels of IL-10 and did not secrete TNF-α and IL-6, suggesting that IL-17 does not affect the secretion of these pro-inflammatory cytokines associated with macrophage differentiation. Our results support previous findings that monocyte differentiation to macrophages by treatment with IL-17 for 6 days did not affect macrophage differentiation at the gene or protein expression level, including IL-6 and TNF-α [17]. By contrast, mature macrophages or monocytes increase TNF-α and IL-6 expression in response to IL-17 [17, 18]; moreover, mature macrophages with M-CSF stimulated with LPS and IL-17 increase TNF-α expression compared with IL-17 alone [17]. In this context, we stimulated macrophages with IL-17 for 6 days in the presence and absence of oxLDL. We noted that macrophages treated with IL-17 and oxLDL did not exhibit an increase in the secretion of IL-10 compared with macrophages treated only with IL-17. However, macrophages treated with IL-17 exhibited drastically increased levels of IL-6 and TNF-α in the presence of oxLDL compared with macrophages cultured only with IL-17. These results suggest that macrophages differentiated only with IL-17 acquire inflammatory capacity in response to oxLDL, similar to M1. These results are supported by in vitro studies showing that exogenous TNF-α activates macrophages only after priming with IFN-γ [32].

## Conclusions

Our study demonstrates that macrophages differentiated with IL-17 do not express differentiation markers or cytokines specific to M1 or M2. However, macrophages differentiated with IL-17 expressed TLR2 and TLR4 and formed few foam cells. In addition, in the presence of oxLDL, macrophages differentiated with IL-17 acquire inflammatory capacity through the secretion of pro-atherogenic cytokines, such as TNF-α, and the expression of costimulatory molecules, similar to M1. These findings suggest that IL-17 may contribute to the differentiation of macrophages, which, in the presence of oxLDL, secrete pro-inflammatory cytokines than contribute to the pathogenesis of atherosclerosis.

## Abbreviations

GM-CSF: Granulocyte macrophage-colony stimulating factor
IFN: Interferon
IL: Interleukin
LPS: Lipopolysaccharide
M1: M1 macrophages
M2: M2 macrophages
M-CSF: Macrophage colony-stimulating factor
oxLDL: Oxidized low-density lipoprotein
PBMCs: Peripheral blood mononuclear cells
PBS: Phosphate-buffered saline
Rh: Recombinant human
Th: T-helper
TLRs: Toll-like receptors
TNF: Tumor necrosis factor
